# CZTSe solar cells prepared by electrodeposition of Cu/Sn/Zn stack layer followed by selenization at low Se pressure

**DOI:** 10.1186/1556-276X-9-678

**Published:** 2014-12-15

**Authors:** Liyong Yao, Jianping Ao, Ming-Jer Jeng, Jinlian Bi, Shoushuai Gao, Qing He, Zhiqiang Zhou, Guozhong Sun, Yun Sun, Liann-Be Chang, Jian-Wun Chen

**Affiliations:** 1 Institute of Photoelectronic Thin Film Devices and Technology and Tianjin Key Laboratory of Thin film Devices and Technology, Nankai University, Tianjin 300071, People’s Republic of China; 2 Department of Electronic Engineering, Chang Gung University, 259, WenHwa 1st Road, KweiShan, 333, Taoyuan, Taiwan

**Keywords:** Cu_2_ZnSnSe_4_ (CZTSe), Electrodeposition, Cu/Sn/Zn precursors, Selenization, Solar cells

## Abstract

Cu_2_ZnSnSe_4_ (CZTSe) thin films are prepared by the electrodeposition of stack copper/tin/zinc (Cu/Sn/Zn) precursors, followed by selenization with a tin source at a substrate temperature of 530°C. Three selenization processes were performed herein to study the effects of the source of tin on the quality of CZTSe thin films that are formed at low Se pressure. Much elemental Sn is lost from CZTSe thin films during selenization without a source of tin. The loss of Sn from CZTSe thin films in selenization was suppressed herein using a tin source at 400°C (A2) or 530°C (A3). A copper-poor and zinc-rich CZTSe absorber layer with Cu/Sn, Zn/Sn, Cu/(Zn + Sn), and Zn/(Cu + Zn + Sn) with metallic element ratios of 1.86, 1.24, 0.83, and 0.3, respectively, was obtained in a selenization with a tin source at 530°C. The crystallized CZTSe thin film exhibited an increasingly (112)-preferred orientation at higher tin selenide (SnSe_*x*_) partial pressure. The lack of any obvious Mo-Se phase-related diffraction peaks in the X-ray diffraction (XRD) diffraction patterns may have arisen from the low Se pressure in the selenization processes. The scanning electron microscope (SEM) images reveal a compact surface morphology and a moderate grain size. CZTSe solar cells with an efficiency of 4.81% were produced by the low-cost fabrication process that is elucidated herein.

## Background

Thin film materials such as copper indium gallium diselenide (CIGS) [1] and cadmium telluride (CdTe) [2] are attracting much attention owing to their potential applications in the harvesting of solar energy. However, restrictions on the usage of the heavy metal Cd and limited supplies of In and Te are projected to limit the fabrication of existing chalcogen-based technologies to <100 GWp annually [3,4]. Low-cost earth-abundant copper-zinc-tin-chalcogenide kesterites, Cu_2_ZnSnS(Se)_4_, and Cu_2_ZnSn(S,Se)_4_ have been studied as potential alternatives to CIGS or CdTe [5-8]. Recently, a liquid-based copper zinc tin sulfur-selenium (CZTSSe) solar cell with an efficiency of 12.6% was developed [5], and a 14-cm^2^ sub cell with an efficiency of over 10.8% has been fabricated [8]. This rapid increase in the efficiency of CZTSSe solar cells signifies their huge potential in the future [5,9-11]. Many methods, including both vacuum and non-vacuum methods, such as sputtering deposition [12], co-evaporation [6,7], solution ink printing [5,9,11], and electrodeposition [13,14], have been used to prepare Cu_2_ZnSnS(e)_4_ layers. Among these methods, electrodeposition has the following advantages: (a) it does not require a vacuum, (b) it is low-cost, (c) it provides thin films that are uniform over a large area, and (d) it enables the accurate control of morphology and composition [15,16]. Unlike the co-electrodeposition of copper-zinc-tin, the electrodeposition of stacked metal layers allows precise control of the quantity deposited and is effective under a large range of deposition conditions of temperature, pH, and concentration of the main salt or addition agent. The morphology of the stacked metal layers can be easily controlled. Post-annealing improves the uniformity of the distribution of elements and promotes the conversion of elemental phases to alloyed phases, which have a significant effect on the formation of CZTSSe thin films [13].

As is well known, a two-step process is widely used to synthesize high-quality CZTS(Se) thin films [17]. In this process, the precursor layer is prepared by vacuum or non-vacuum methods and then thermal annealing is performed at high sulfur or selenium pressure [7,13]. However, selenization at high S(Se) pressure is likely to form a thick molybdenum disulfide/diselenide (MoS(Se)_2_) layer [5,7,13,14]. An excessively thick MoS(Se)_2_ layer degrades the performance of the device [18,19]. Therefore, treatment under low Se pressure has been suggested to prevent the formation of such a thick molybdenum diselenide (MoSe_2_) layer. However, the potential loss of the Sn metal from Cu_2_ZnSnSe_4_ (CZTSe) thin films at high temperature raises the additional difficulty of controlling the composition and phase of the films. Adding tin selenide (SnSe_*x*_) gas during annealing can inhibit the decomposition of CZTSe thin films [20]. This work studies the use of a low Se pressure [21] and additional SnSe_*x*_ vapor in the preparation of high-quality CZTSe thin films.

## Methods

**Figure 1:**
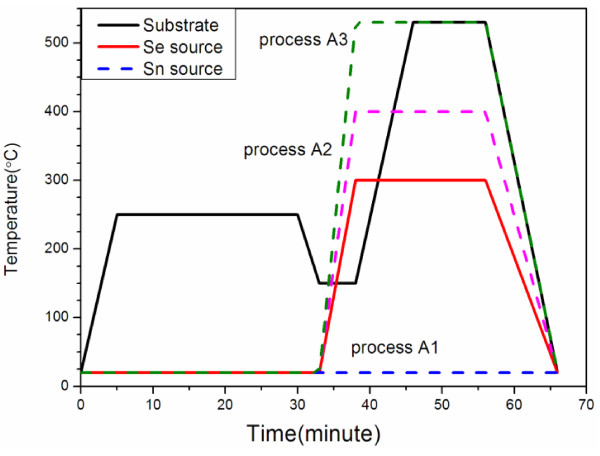
**Three selenization processes.** Without a tin source (A1), with a tin source at a temperature of 400°C (A2), and with a tin source at 530°C (A3).

**Figure 2:**
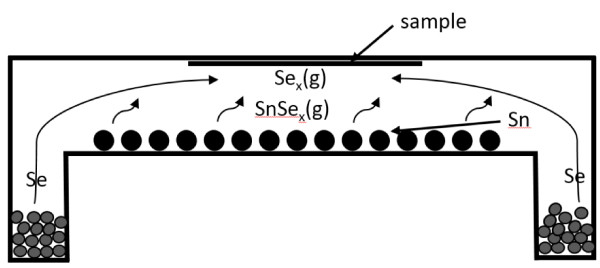
The schematic diagram of the reaction from a tin source with Se.

Electrodeposited thin metal stacks were used to fabricate CZTSe thin films by selenization. The selenization process was performed in an environment with a low selenium pressure of 48.5 Pa, which was set by controlling the temperature of the selenium source. The tin concentration was controlled by varying the temperature of the tin source. Three processes were studied: process A1 involved no tin source, process A2 involved a tin source at 400°C, and process A3 involved a tin source at 530°C. The cathode in the electrodeposition process was a 4 × 4 cm^2^ piece of soda-lime glass on which a 1,200-nm-thick double Mo layer had been deposited by DC-magnetron sputtering. The metal stacks of copper, tin, and zinc were electrodeposited on a molybdenum layer by the constant current method using a three-electrode system at room temperature. The stacking sequence of the three metals was copper/tin/zinc (Cu/Sn/Zn) with the Cu layer at the bottom and adjacent to the Mo layer, the Sn layer was in the middle, and the zinc layer was on the top surface. The initial molar ratios of the electroplated metals were Zn/Cu = 0.67 and Sn/Cu = 0.53. The ratio of Cu, Sn, and Zn was controlled by adjusting the electroplating times of copper, tin, and zinc. The reference electrode and anode were electrically linked to each other. Tin was electrodeposited from a commercially available electrodeposition bath, which contained mesylate, tin methanesulfonate, and additives RX-851 from Rongxing Electronics (Zhejiang, China). Copper was electrodeposited from a laboratory-made solution and contained 187.5 g/L of sulfate pentahydrate. The solution of zinc was developed in-house using 0.2 M zinc vitriol that was dissolved in 0.5 M methane sulfuric acid [22]. The pH of the solution was adjusted to 2.0 by adding sodium hydroxide. The copper, tin, and zinc layers were electrodeposited using direct current with current densities of 50, 3, and 20 mA/cm^2^, respectively. The typical deposition time was approximately 100 s. The electrodeposited metal stacks were annealed at 250°C in inert gas at 1,000 Pa and subsequently underwent those three aforementioned selenization processes, as presented in Figure 1. Figure 2 schematically depicts the reaction of a tin source with Se. The SnSe_*x*_ that is required to form stoichiometric CZTSe thin films was supplied by the reaction of a tin source with Se. A CZTSe solar cell with a Mo/CZTSe/CdS/i-ZnO/Al-ZnO/Ni-Al structure was fabricated with an active area of 0.358 cm^2^.

The composition of the CZTSe thin film was obtained using a Magix(PW2403 (PANalytical LTD., the Netherlands)) X-ray fluorescent spectrometer (XRF) with an Rh-anode, which was calibrated by inductively coupled plasma spectroscopy (ICP). The structures of the selenized samples were elucidated using a Philips X-pert Pro diffractometer (PANalytical Ltd., the Netherlands) with Cu radiation and a Renishaw inVia Raman spectroscope (Renishaw Ltd., UK). Surface and cross-sectional observations were made using a scanning electron microscope (SEM, JEOL JSM-6700 (JEOL Ltd., Akishima-shi, Japan)). The depth profiles of the elements were obtained by secondary ion mass spectroscopy (SIMS, IMS-4 F, CAMECA, Nancy, France). Current–voltage (J-V) measurements of CZTSe solar cells were made under illumination by a standard AM1.5 spectrum of 100 mW/cm^2^ at room temperature using a constant-light solar simulator, which was calibrated using a standard monocrystalline Si solar cell.

## Results and discussion

**Figure 3:**
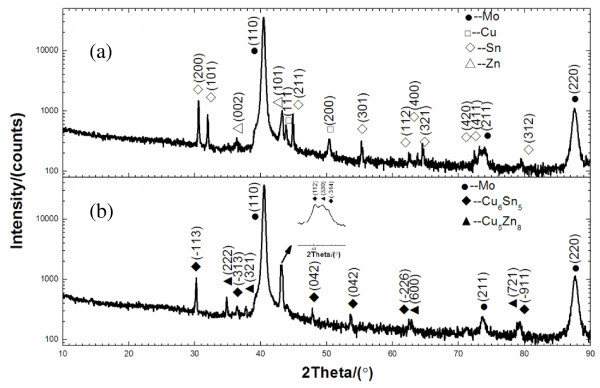
The XRD spectra of the electrodeposited Cu/Sn/Zn metal layer (a) before and (b) after thermal annealing.

Figure 3 presents the X-ray diffraction (XRD) spectra of the electrodeposited Cu/Sn/Zn metal layer before and after thermal annealing. The figure reveals that the electrodeposited metal stack layer comprised elemental phases of copper, tin, and zinc. However, 30 min of annealing at 250°C in inert gas at a pressure of 1,000 Pa caused dramatic changes. The binary alloy of copper/tin (Cu_6_Sn_5_) and copper/zinc (Cu_5_Zn_8_) is the main phases in Figure 3b, revealing that a uniform Cu/Sn/Zn layer was obtained. Heat treatment at 250°C mixed the Cu, Sn, and Zn metal layers to form only Cu_6_Sn_5_ and Cu_5_Zn_8_ alloy phases, and no elemental metallic phase was observed, indicating that the Cu, Sn, and Zn elements had become intermixed and uniformly distributed throughout the Cu/Sn/Zn layer. A uniform distribution of copper favors the adhesion of the CZTSe layer to the Mo surface following selenization, and a uniform distribution of Cu/Sn/Zn favors the formation of CZTSe thin films [13,23].

Table 1 shows the composition of the CZTSe thin films that were prepared by various selenization processes. The use of a tin source in the selenization process under low Se (48.5 Pa) pressure significantly influences the composition of CZTSe thin films. Without a tin source, the CZTSe thin films have a poor stoichiometric ratio and suffer a severe loss of Sn. With a tin source, CZTSe thin films are obtained with a near-stoichiometric composition. During the selenization process, tin may undergo many reactions, including the evaporation of tin and the formation of tin(II) selenide (SnSe) or tin(IV) diselenide (SnSe_2_), as follows.

**Table 1:** The composition of the CZTSe thin films prepared by various selenization conditions of A1, A2, and A3

**Sample**	**Cu**	**Zn**	**Sn**	**Se**	**Cu/(Zn + Sn)**	**Zn/Sn**
**A1**	26.03	15.37	8.24	50.36	1.10	1.86
**A2**	22.81	15.38	11.55	50.27	0.85	1.33
**A3**	22.63	15.14	12.19	50.04	0.83	1.24

(1) $$Sn\left(s\right)\to Sn\left(g\right)$$

(2) $$Sn\left(s\right)+1/\text{xSex}\left(g\right)\to \text{SnSe}\left(s\right)$$

(3) $$\text{SnSe}\left(s\right)\to \text{SnSe}\left(g\right)$$

(4) $$Sn\left(s\right)+2/\text{xSex}\left(g\right)\to SnSe_{2}\left(s\right)$$

(5) $$SnSe_{2}\left(s\right)\to SnSe_{2}\left(g\right)$$

**Figure 4:**
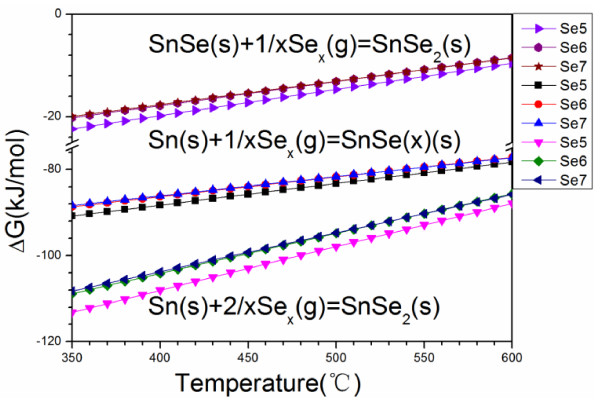
**Gibbs energy change of the reaction of Sn with Se**_***x ***_**as the main reactant.** The Gibbs energy change of the reaction of Sn with Se_*x*_ (where *x* = 5, 6, 7) as the main reactant at a temperature of less than 550°C.

Figure 4 presents a change in the Gibbs energy upon the reaction of Sn with Se_*x*_ (where *×* = 5, 6, 7) as the main reactant, at a temperature below 550°C [24]. The change in the Gibbs energy upon the reaction of Sn with Se_*x*_ was calculated using the Gibbs free energy data from Binnewies’s and Milke’s book [25]. The changes in the Gibbs energy that are associated with the reaction of Sn with Se to form SnSe or SnSe_2_ are negative, indicating that this reaction occurs spontaneously at 400°C or 530°C. The saturated vapor pressures of SnSe, SnSe_2_, and Sn are calculated using the following equations based on data from [21,26].

(6) $$\text{SnSe}:\cdot \log\;P\;=-\frac{10746}{T}+7.676\left(\text{Pressure}\;\text{unit}:\text{bar}\right)$$

(7) $$SnSe_{2}:\cdot \log\;P=-\frac{9670}{T}+8.03\left(\text{Pressure}\;\text{unit}:\text{bar}\right)$$

(8) $$\begin{array}{c} Sn:\cdot \log\;P=-118.452+\frac{13744}{T}\\ \;+6.4004\text{logT}-0.00097861T-4.2795\\ \;\times 10^{-10}T^{2}\left(\text{Pressure}\;\text{unit}:\text{bar}\right) \end{array}$$

**Figure 5:**
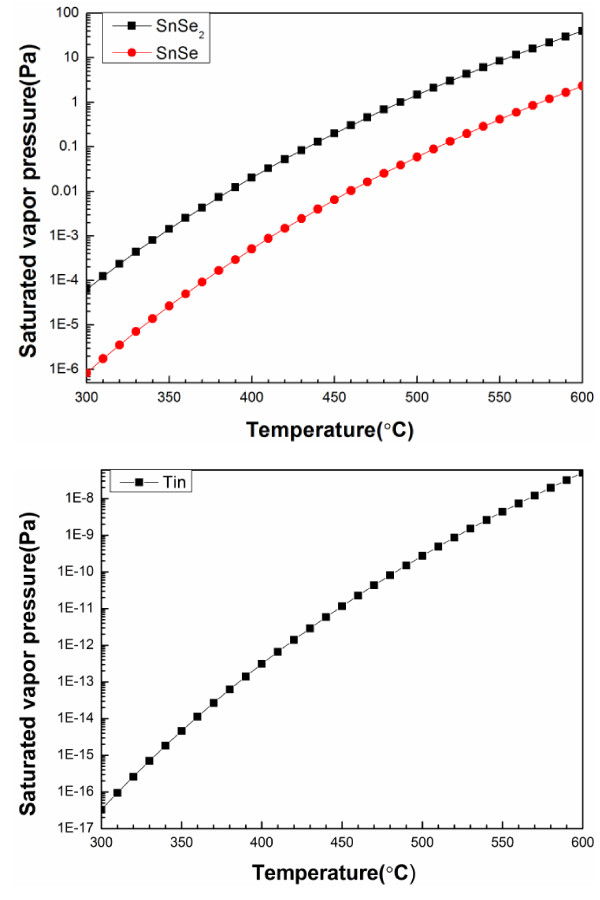
**The saturated vapor pressures of SnSe, SnSe**_**2**_**, and tin.**

Since the saturated vapor pressures of SnSe and SnSe_2_[26] considerably exceed that of Sn [21], as presented in Figure 5, the evaporation of Sn can be neglected, whereas that of SnSe_*x*_, which proceeds at a considerable rate, cannot. Consequently, the constituents of the selenization atmosphere are Se_*x*_ (*x* = 2, 3,…, 7), SnSe, and SnSe_2_. Equation 9 presents the instability of CZTSe thin films at high temperature [20,27]. CZTSe films are well known to decompose rapidly at temperatures of greater than 400°C [11-13]. SnSe and Se evaporate, leaving copper selenide (Cu_*x*_Se) and zinc selenide (ZnSe) [17]. According to the Arrhenius equation, the chemical reaction rate increases exponentially with temperature. The CZTSe decomposes rapidly at the temperature of 530°C, which greatly exceeds 400°C.

(9) $$\begin{array}{c} Cu_{2}\text{ZnSnS}e_{4}\to Cu_{2}Se+\text{ZnSe}+\text{SnSe}\left(g\right)\\ \;+1/2Se_{2}\left(g\right) \end{array}$$

**Figure 6:**
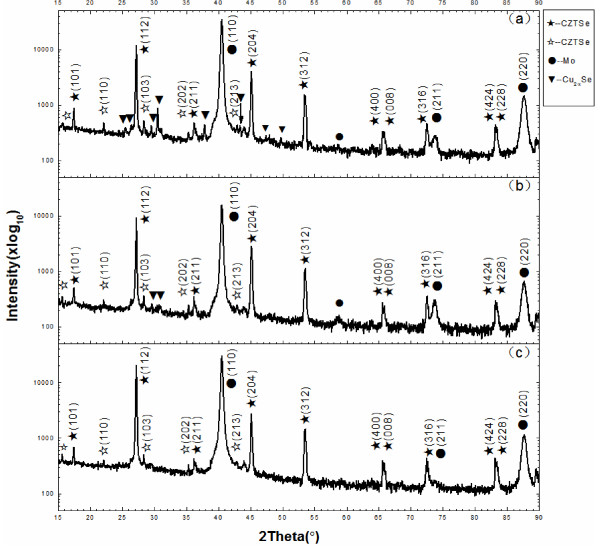
**The X-ray diffraction spectrum of CZTSe thin films that are prepared under three selenization conditions. (a)** Without a tin source (A1), **(b)** with a tin source at 400°C (A2), and **(c)** with a tin source at 530°C (A3).

Figure 6 presents the X-ray diffraction spectrum of CZTSe thin films that is prepared by various selenization processes. The CZTSe films decompose into binary phases of Cu_x_Se, ZnSe, and SnSe at higher temperatures. Since the ZnSe phase has similar X-ray diffraction peaks to those of the CZTSe films, distinguishing between the two is difficult. Additionally, the SnSe phase evaporates easily at high temperature. Therefore, the Cu_x_Se phase is the only evidence of the decomposition of the CZTSe films. The CZTSe films that are prepared under low SnSe_*x*_ pressure yield a clear Cu_2-x_Se peak in Figure 6a,b. However, the CZTSe films that are prepared at high SnSe_*x*_ pressure exhibit no such peak in Figure 6c, indicating that high SnSe_*x*_ partial pressure suppresses the evaporation of the SnSe, and it may suppress the decomposition of the CZTSe films. Following selenization without a tin source (A1), the CZTSe thin film yields a main peak with a (112) preferred orientation, and the presence of the Cu_2-x_Se phase is consistent with the XRF results, which are presented in Table 1 and reveal that the film is Cu-rich. A main (112) diffraction peak is observed following selenization with a tin source at 400°C (A2), which also yields a weak (204) diffraction peak and much weaker Cu_2-x_Se diffraction peaks. Following selenization with a tin source at 530°C (A3), a main (112) diffraction peak is also observed but no Cu_2-x_Se-related peak is observed. Table 2 lists the intensities of the XRD peaks of CZTSe thin films that were prepared using various selenization processes. Processes A1 and A2 yield (112) diffraction peaks of equal intensity, but the A2 process yields a much weaker (204) diffraction peak. The (204) crystallization peak of CZTSe becomes weaker when the reaction of Sn with Se_*x*_ introduces SnSe_*x*_ (*x* = 1, 2) into the selenization atmosphere. The (204) diffraction peak obtained following the A3 process is smaller than that obtained following the A2 process. A higher partial pressure of SnSe_*x*_ was obtained at a higher tin source temperature, resulting in weaker crystallinity of the CZTSe (204) plane. The intensity ratios I(112)/I(204)following the A1, A2, and A3 processes were 3.08, 3.84, and 8.40, respectively, revealing that a higher SnSe_*x*_ partial pressure yielded the preference of the CZTSe thin films for the (112) orientation. The CZTSe films were unstable at high temperature especially at low pressure. Different preparation processes may result in different preferred orientations because of the different chemical potentials in the experimental atmosphere. The CZTSe films may exhibit a (112)-preferred orientation when the chemical potential of the CZTSe films equal the experimental atmosphere. Equilibrium between the chemical potential of the experimental atmosphere and the CZTSe film can be achieved. The decomposition reaction of the CZTSe films is reversible. A sufficiently high partial pressure of SnSe_*x*_ in the experimental atmosphere promotes the reverse of the decomposition of the CZTSe films, which is their formation. A thermodynamic equilibrium condition exists at a sufficiently high SnSe_*x*_ partial pressure. The CZTSe films are stable and exhibit a (112)-preferred orientation. Restated, a high SnSe_*x*_ partial pressure suppresses the evaporation of SnSe, potentially suppressing the decomposition of the CZTSe films, causing the films to adopt a (112)-preferred orientation. Interestingly, in Figure 6, a large Mo peak is observed but no MoSe_2_ peak is observed, probably owing to the use of a low Se pressure (300°C) in this experiment. According to ICSD16948, the peak at 31.70° corresponds to (101) and that at 55.92° corresponds to (110). In the literature, a high Se pressure is commonly used in the selenization process. A temperature of greater than 500°C corresponds to a pressure of over 7,281 Pa.

**Table 2:** The intensities of the XRD (112) and (204) peaks of CZTSe thin films prepared by various selenization conditions

**Sample**	**I(112)/counts**	**I(204)/counts**	**I(112)/I(204)**
A1	10,993	3,571	3.08
A2	10,308	2,685	3.84
A3	17,896	2,131	8.40

**Figure 7:**
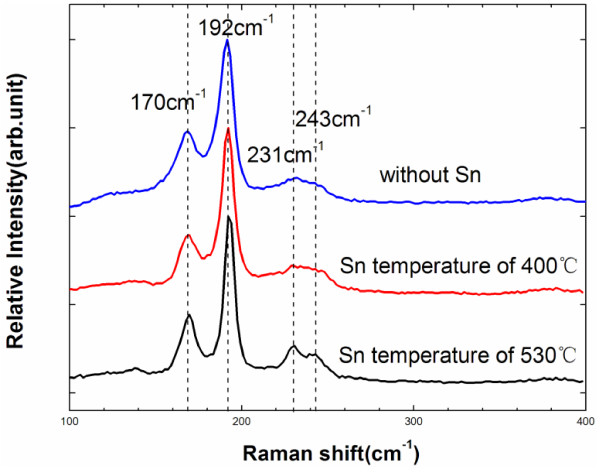
**The Raman spectra of CZTSe thin films prepared by the various selenization conditions.** (Blue) without a tin source, (red) with a tin source at 400°C, and (black) with a tin source at 530°C.

Owing to the limitations of the XRD characterization of CZTSe thin films, Raman spectroscopy was used to clarify the above phase analysis. Figure 7 shows the Raman spectra of CZTSe thin films that were prepared using various selenization processes without a tin source (blue), with a tin source at 400°C (red), and with a tin source at 530°C (black). Three Raman shift peaks at 170, 192, and 231 cm^-1^ are consistent with the results of previous studies. A weak peak between 231 and 253 cm^-1^ is also observed, consistent with monograin powder samples [28]. No peak that is related to a secondary phase, such as Cu_2_Se, SnSe, or Cu_2_SnSe_3_, is observed.

**Figure 8:**
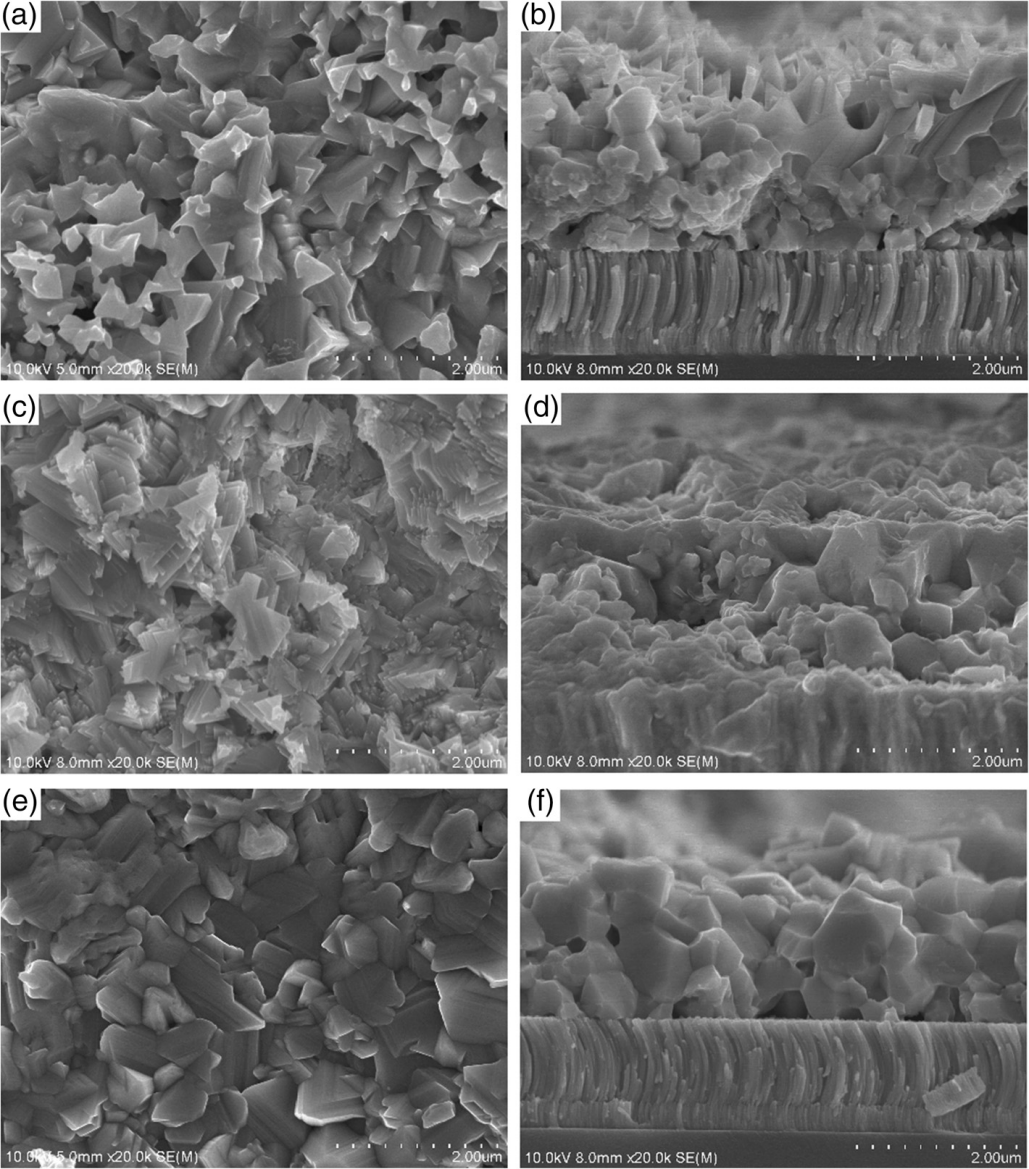
**Surface and cross-section SEM images of CZTSe thin films prepared by the various selenization conditions. (a-b)** Without a tin source (A1), **(c-d)** with a tin source at a temperature of 400°C (A2), and **(e-f)** with a tin source at 530°C (A3).

Figure 8 presents the surface and cross-section SEM images that further elucidate the microstructure. Figure 8a,b presents the surface and cross-section SEM images of CZTSe thin films that were selenized without a tin source. The thickness of the CZTSe layer was approximately 1.5 μm, and the structure of the film surface was granular and loose. Figure 8c,d presents the surface and cross-section SEM images of CZTSe thin films that were selenized with a tin source at 400°C. The addition of a tin source increased the size of the grains in the CZTSe thin film and caused small grains to form at its surface. The cross-sectional view reveals greatly improved crystallization with compact organization and larger grains when the tin source is used. No pinhole is observed at the interface between the absorber layer and the Mo back contact. Figure 8e,f presents the surface and cross-section SEM images of the CZTSe thin films that were selenized with a tin source at 530°C. As presented in Figure 8e, the surface morphology was greatly improved. A compact surface and large grains are observed. At low Se pressure without the supply of any SnSe_*x*_, CZTSe thin films decompose rapidly, yielding a poor surface and causing internal crystallization. At low Se pressure with a tin source at 400°C, the partial pressure of SnSe partially inhibits the internal decomposition of CZTSe. Favorable internal but poor surface thin film morphologies are obtained. At low Se pressure with a tin source at 530°C, the partial pressure of SnSe_*x*_ suffices to inhibit the surface and inner decompositions of the CZTSe thin films, greatly improving both the quality of the surface and the microstructure of the films. This result is consistent with the XRF and XRD measurements. The introduction of SnSe_*x*_ into the selenization atmosphere by the reaction of Sn with Se considerably increased the controllability of the composition and the crystallization of the CZTSe thin film. From Figure 8b,d,f, the size of the grains close to the Mo back contact was close to that of the interior grains. No MoSe_2_ is observed, consistent with the XRD results. Therefore, we believe that the MoSe_2_ layer between CZTSe and the Mo layer either does not exist or is very thin.

**Figure 9:**
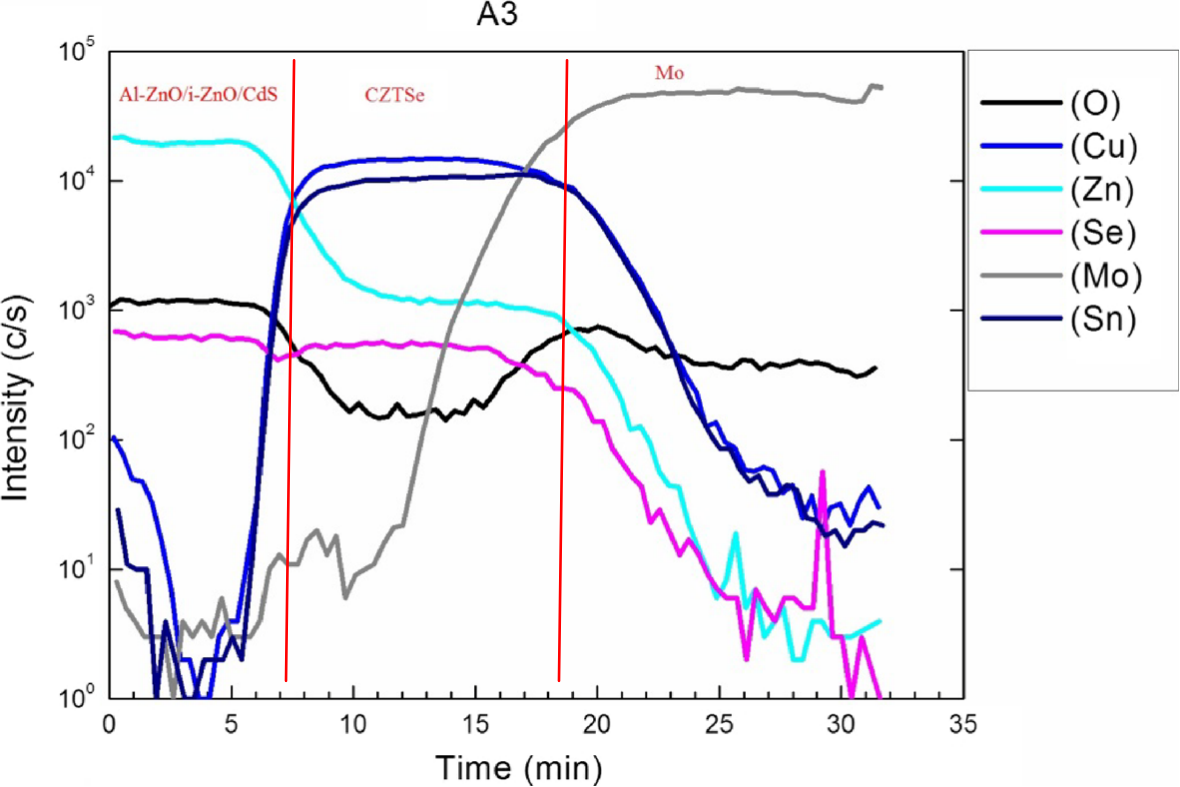
SIMS profiles of the CZTSe thin film prepared by the selenization process A3.

**Figure 10:**
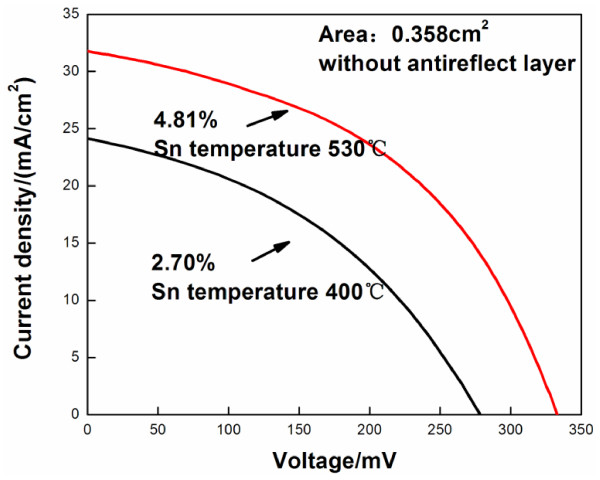
Current–voltage curves of CZTSe solar cells selenized by the A2 and A3 processes.

To validate the above observations, SIMS measurements were made on the CZTSe thin film as it underwent process A3. Figure 9 presents the SIMS profiles of the CZTSe solar cell with the structure Al-ZnO/i-ZnO/CdS/CZTSe/Mo. In the first 6 or 7 min, the SIMS profiles include signals associated with Al-ZnO and i-ZnO layers. The Zn signal is strong, and the Cu and Sn signals are weak. From 10 to 20 min, the SIMS profiles include a signal from the CZTSe layer. The four elements of copper, zinc, tin, and selenium are uniformly distributed. The SIMS result reveals that the CZTSe film has a uniform composition. The scanned elements were Cu, Zn, Sn, Se, and O; the scanning rate was approximately 100 nm/min, and the signal collection rate was 2.5 times per minute, so the accuracy of the measured depth of each scanned element was approximately 40 nm. The oxygen content of the film was high, similar to that used in Guo’s paper [22], perhaps because the Cu/Sn/Zn metal layer was electrodeposited in a non-vacuum. The changes in the intensities of the Cu, Zn, Sn, and Se signals were consistent with each other, revealing that the four elements were similarly distributed. Additionally, when a thick MoSe_2_ layer was present at the interface between Mo and CZTSe, the change in the intensity of the Se signal from MoSe_2_ exceeded that of the Se signal from CZTSe and the intensities of the Cu, Sn, and Zn signals changed only slightly. Fortunately, this strengthening of the Se signal was not observed in the SIMS figure, except for one increase in the signal intensity at 29 min, perhaps caused by the difference between the degrees of crystallization of the double layer of Mo that was caused by the changes in the preparation of the double layer of Mo. The interface of the double layer of Mo attracts Se, which accumulates there. The intensity of the Mo signal was similar to that of the Mo signal in the CIGS/Mo sample that was prepared in the same laboratory by electrodeposition followed by selenization [29]. We conclude that either no MoSe_2_ layer was present between Mo and the CZTSe layer, or such a layer was present but thinner than the resolution of the measuring instrument. Figure 10 plots the current–voltage curves of CZTSe solar cells that were selenized by the A2 and A3 processes, which yielded CZTSe solar cells with the efficiencies of 2.7% and 4.8%, respectively. The efficiency of the solar cell increased markedly with the Sn temperature. The A3 process yielded the higher fill factor, short-circuit current, and open-circuit voltage. However, the fill factor obtained herein was lower than those obtained by other groups, mainly because the low selenization temperature that was used herein resulted in poor crystallinity and a rough surface of the electrodeposited stack metal layer.

## Conclusions

Preparing high-quality CZTSe thin films under low selenium pressure is difficult owing to severe Sn loss. The presence of SnSe_*x*_ is critical to the formation of stoichiometric CZTSe thin films, especially when selenization is performed at high temperature and low Se pressure. A higher SnSe_*x*_ partial pressure yields better crystallinity of CZTSe with a preferred (112) orientation. A very thin MoSe_2_ layer may be present at the interface between Mo and the CZTSe layer following selenization at a low selenium pressure and a substrate temperature of 530°C. CZTSe solar cells with an efficiency of 4.81% are formed by the low-cost electrodeposition of a Cu/Sn/Zn stack layer followed by selenization at a low Se pressure.

## Competing interests

The authors declare that they have no competing interests.

## Authors’ contributions

LY carried out the electrodeposition of the stack metal layer and selenization and drafted the manuscript. JA carried out the design of electrodeposition, coordination, and helped to draft the manuscript. M-JJ carried out the discussion of experimental result and helped to draft the manuscript. JB carried out the Mo deposition by sputter. SG carried out ZnO and AZO depositions by sputter. QH carried out the metallization for solar cells. ZZ carried out the characterization of CZTSe thin films. GS carried out the CdS deposition by CBD and the performance measurement of solar cells. YS participated in the discussion of experimental results and coordination. L-BC participated in the discussion of experimental results. J-WC carried out device characterizations. All authors read and approved the final manuscript.

## Authors’ information

LY is a Ph.D student in the Institute of Photoelectronic Thin Film Devices and Technology, Nankai University, China.

JA is a professor in the Institute of Photoelectronic Thin Film Devices and Technology, Nankai University, China.

M-JJ is a professor in the Department of Electronic Engineering in Chang Gung University, Taiwan.

JB is a Ph.D student in the Institute of Photoelectronic Thin Film Devices and Technology, Nankai University, China.

SG is a senior engineer in the Institute of Photoelectronic Thin Film Devices and Technology, Nankai University, China.

QH is a senior engineer in Institute of Photoelectronic Thin Film Devices and Technology, Nankai University, China.

ZZ is a senior engineer in the Institute of Photoelectronic Thin Film Devices and Technology, Nankai University, China.

GS is a senior engineer in the Institute of Photoelectronic Thin Film Devices and Technology, Nankai University, China.

YS is a professor in Institute of Photoelectronic Thin Film Devices and Technology, Nankai University, China.

L-BC is a professor in the Department of Electronic Engineering in Chang Gung University, Taiwan.

J-WC is a master student in the Department of Electronic Engineering in Chang Gung University, Taiwan.
